# Targeting Protein Tyrosine Phosphatases via PROteolysis-TArgeting Chimeras (PROTACs): Current Developments and Prospects

**DOI:** 10.3390/molecules30224449

**Published:** 2025-11-18

**Authors:** Rosanna Maccari

**Affiliations:** Department of Chemical, Biological, Pharmaceutical and Environmental Sciences, University of Messina, Viale F. Stagno d’Alcontres 31, 98166 Messina, Italy; rmaccari@unime.it

**Keywords:** PROTACs, targeted protein degradation, protein tyrosine phosphatases, diabetes, cancer

## Abstract

Protein tyrosine phosphatases (PTPs) are prominent enzymes which play pivotal roles in the regulation of multifarious cellular functions. Dysregulations of several PTPs have well-documented implications in the pathogenesis of various human diseases, including diabetes, cancer, and neurodegenerative and inflammatory disorders. Therefore, PTPs are considered attractive targets for therapeutic intervention. However, the development of novel drugs targeting these enzymes has encountered several difficulties. Currently, it has become clear that improving PTP druggability can be an attainable goal, through different medicinal chemistry approaches. Besides the development of allosteric inhibitors of PTPs, the design of PROteolysis-TArgeting Chimeras (PROTACs) has emerged as a promising strategy capable of providing a useful alternative mechanism to control these enzymes through their targeted degradation. Although the development of PROTACs directed to PTPs is still in its infancy, the results so far available are promising; this perspective study focuses on this class of potential novel drugs, highlighting advantages and challenging aspects to consider for future progress.

## 1. Introduction

The design of PROteolysis-TArgeting Chimeras (PROTACs) has recently emerged as an innovative strategy to discover agents capable of targeting proteins implicated in the development of different human pathologies. The PROTAC approach is based on the mechanism of cellular protein degradation regulated by the Ubiquitin Proteasome System (UPS) [1,2,3]. UPS is crucial to maintain cellular homeostasis in response to different stimuli, such as increased oxidative stress, heat shock and DNA damage, which can cause intracellular accumulation of misfolded and unfolded proteins [1]. These abnormal proteins can be toxic for cells and, therefore, they are quickly degraded and eliminated as inactive oligomers. UPS-mediated protein degradation is a multistep process triggered by the activation of ubiquitin, a small cellular protein which is transferred to lysine residues of the target protein. The process requires the presence of three types of enzymes, i.e., E1 (ubiquitin-activating enzyme), E2 (ubiquitin-conjugating enzyme), and E3 (ubiquitin ligase). E1 enzyme activates ubiquitin by converting it into a high-energy thioester, by means of ATP hydrolysis; the active intermediate is then transferred to an E2 conjugating enzyme. In turn, this latter interacts with an E3 ligase, which is responsible for both the recruitment of a specific protein as substrate and the final transfer of ubiquitin from the E2-thioester complex to the target, thus determining the specificity of the whole process. After multiple rounds, the polyubiquitination of the substrate results in the recognition of the tagged protein by the proteasome 26S, which finally degrades the ubiquitinated protein by converting it into short inactive peptides [1].

PROTACs are rationally designed as heterobifunctional ligands capable of binding simultaneously both an E3 ligase and a protein of interest (POI) that must be degraded, being implicated in the pathogenesis of a certain disease. Therefore, the structure of a PROTAC is schematically formed by two binding moieties, i.e., a ligand (“warhead”) targeting a POI and a ligand able to recruit an E3 ligase (E3 ligase binder), which are tethered together by a suitable linker [4,5,6,7]. Through these two functional portions, a PROTAC molecule can form a POI/PROTAC/E3 complex in which the POI and E3 ligase are in proximity. The formation of this ternary complex can facilitate the ubiquitination of the POI, which subsequently is recognized and destroyed by the proteasome, while the PROTAC is released intact and can be reused repeatedly to form other POI/PROTAC/E3 complexes [4,5,6,7,8]. This “pseudo-catalytic” action is an attractive feature of PROTACs, because potentially it can lead to potent prolonged efficacy at low concentrations, possibly resulting in improved safety [2,3,4,5].

For the last few years, this drug design approach has attracted great interest, since PROTACs could provide alternative or complementary pharmacological tools, compared with conventional enzyme inhibitors or receptor antagonists, by specifically degrading proteins that are overexpressed or deregulated in pathogenic processes [4,5,9]. In some cases, structural features of the protein binding site may hamper the design of ligands capable of establishing efficient interactions with the target and, at the same time, endowed with appropriate drug-like properties. This could lead to some proteins considered as challenging, or even “undruggable”, targets for pharmacological intervention. However, a ligand that does not display sufficient effectiveness or adequate profile to be further developed as a drug might be exploited for design of PROTACs. These heterobifunctional degraders do not require the binding to the active site or to specific allosteric regions of a POI, but potentially any site of the protein could be targeted. Therefore, a wide number of compounds with different physico-chemical and structural features could be converted into suitable warheads, thus potentially extending the chemical space in which compounds of medicinal interest may be identified. At the same time, the PROTAC approach could considerably extend the druggable proteome. In fact, the targeting of different proteins, including transcription factors, scaffolding proteins and aggregated proteins, as well as proteins belonging to families characterized by high homology in the binding sites, such as kinases and phosphatases, might take advantage from this approach [2,3,10,11].

At present, the clinical evaluation of promising PROTAC candidates is ongoing at various stages, but no drug acting by means of this mechanism was approved for therapeutic use so far. Most PROTACs currently in clinical development are indicated for different types of cancer, being directed to POIs that are dysregulated in human tumors, such as kinases and steroid receptors [3]. However, a huge variety of proteins might be targeted by PROTACs, possibly providing novel therapeutic opportunities for numerous diseases, besides cancer [5,10,12].

Recently, protein tyrosine phosphatases (PTPs) emerged as a new class of POIs for the design of PROTACs. PTPs are enzymes critically involved in the control of protein tyrosine phosphorylation, by functioning coordinately with protein tyrosine kinases (PTKs). Reversible protein phosphorylation is a dynamic process which is fundamental to maintain cellular homeostasis and to regulate multifarious cell functions, such as proliferation, differentiation, migration, gene expression, signal transduction, survival, immune response [13,14]. It was demonstrated that dysregulations of the activity or expression of certain PTPs are crucially implicated in the development of serious human pathologies, including cancer, autoimmune, metabolic and cardiovascular diseases; therefore, these enzymes have received attention as novel drug targets [13,15,16,17]. However, the discovery of PTP inhibitors suitable as drug candidates has turned out to be a challenging task that requires considerable efforts, especially as compared with the development of PTK inhibitors which, in the last twenty-five years, led to dozens of anticancer agents already approved or under clinical evaluation. Many active site-directed inhibitors of PTPs have been reported in the last two decades, but they often displayed specificity and bioavailability drawbacks which hindered their further development; these failures are mainly related to structural requirements depending on typical features of the catalytic domains of PTPs, such as the presence of highly conserved and polar amino acid residues [14,15,17,18]. Two major alternative approaches have shown promise to obtain molecules with better profiles as potential drugs directed to PTPs: (a) allosteric inhibition, that can lead to the identification of small-molecule inhibitors endowed with more suitable drug-like features, being directed to binding sites which are lined with non-conserved and less polar amino acid residues [17,19,20,21]; (b) PROTAC design, which could offer novel opportunities to overcome the difficulties related to the structural features of PTPs. Besides a brief outline of benefits and limitations of PROTAC approach, this Perspective will focus on the current state-of-the-art of PROTACs targeting PTPs as potential drug candidates, also considering possible future directions and progress.

## 2. The PROTAC Approach: New Opportunities in Drug Design and Discovery

**Figure 1:**
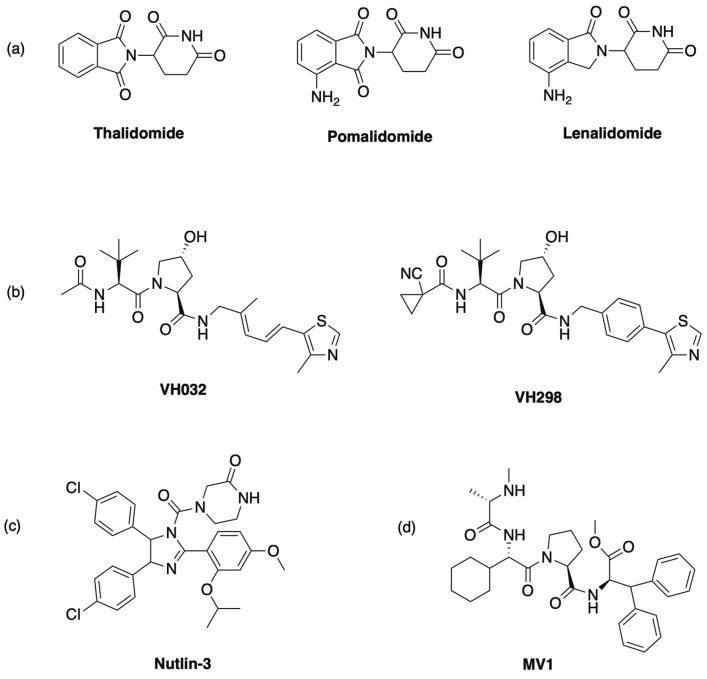
Representative ligands of (**a**) CRBN, (**b**) VHL, (**c**) MDM2 and (**d**) IAPs.

The concept of PROTAC was described for the first time in 2001, when a bifunctional molecule capable to bind simultaneously the methionine aminopeptidase-2 (MetAP2) and the E3 ubiquitin ligase Skp1-Cullin-F(SCF)-β-TRCP and, consequently, to cause the polyubiquitination of MetAP2, was reported [22]. However, the moiety binding SCF-β-TRCP was a phosphopeptide formed by 10 amino acids, because small-molecule ligands for E3 ligases were not known yet. In the following years, E3 ligase ligands endowed with more suitable drug-like properties were developed, thus significantly improving the feasibility of PROTAC-based drug design. In particular, the discovery that the immunomodulatory imide drugs thalidomide, lenalidomide and pomalidomide (Figure 1) can target cereblon (CRBN), which is the substrate-recognition subunit of the cullin-4-containing E3 ubiquitin ligase complex (CRL4) [23,24], paved the way to the design of numerous PROTAC agents. The structure of the co-crystalized thalidomide/CRBN complex (PDB code: 4CI1) provided crucial knowledge; in particular, the interactions of the glutarimide moiety of thalidomide with the residues His380 and Trp382 of CRBN, along with a water-mediated hydrogen bonding between a carbonyl group of the phthalimide portion and His359, were shown to play an important role in determining high affinity of the ligand for this E3 ligase [23]. Interestingly, the benzene ring of the phthalimide moiety was shown to be exposed to the solvent, suggesting that it can be functionalized to tether a linker or to obtain new thalidomide derivatives as E3 binders endowed with improved effectiveness [23].

Besides CRBN binders, small molecules were also identified as ligands directed to different E3 ligases, such as von Hippel–Lindau (VHL) E3 ubiquitin ligase and, subsequently, mouse double minute 2 homolog (MDM2) and inhibitors of apoptosis proteins (IAPs) (Figure 1) [4,8,24,25,26,27]. The discovery of different E3 ligase binders offered further opportunities for PROTAC design by modulating potency, selectivity and drug-like profiles. However, most PROTACs reported so far are directed to CRBN and VHL ligases.

### 2.1. Development of PROTACs as Potential Drugs

**Figure 2:**
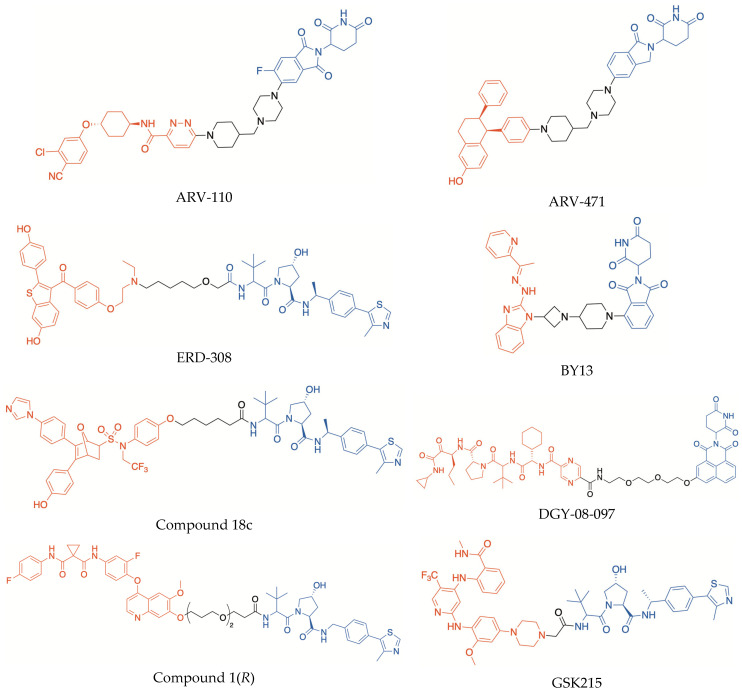
Structures of some representative CRBN- and VHL-recruiting PROTACs (in red, POI-binding moiety; in blue, E3 ligase-binding moiety).

For the last few years, the search for PROTACs as potential novel drugs has attracted considerable attention, and numerous examples of agents based on this approach are present in the recent literature. The structures of some representative PROTACs incorporating CRBN and VHL binder moieties are depicted in Figure 2. Significantly, two of them, i.e., the orally bioavailable agents ARV-110 (bavdegalutamide) and ARV-471 (vepdegestrant), are promising agents that are currently at an advanced stage of clinical evaluation (e.g., phase II clinical trials NCT03888612 and NCT04072952, respectively). ARV-110 targets the androgen receptor and has been evaluated in patients with metastatic castration-resistant prostate cancer. ARV-471 is directed to estrogen receptors (ER) and has been evaluated, alone and in combination with palbociclib, in patients with ER+/HER2- locally advanced or metastatic breast cancer. Furthermore, the combination ARV-471/palbociclib entered a phase III clinical trial (NCT05909397). Interestingly, in both ARV-110 and ARV-471 structures, the warhead is conjugated with the imide–CRBN binder by means of the same 1-(piperidin-4-ylmethyl)piperazine moiety, which was shown to contribute critically to making the hydro-lipophilic balance and pharmacokinetics appropriate for clinical studies [5].

The urgent need for improved ERα degraders to treat ER-positive breast cancer, particularly when conventional endocrine therapies are not effective, prompted the search for several other PROTACs directed to this target. Out of them, ERD-308 (Figure 2) is a promising compound, which was obtained by connecting raloxifene and a VHL binder by means of a flexible methyl pentyl ether linker; this PROTAC displayed potent ERα degrading activity at nanomolar concentrations, proving to be more effective than fulvestrant in degrading ER and inhibiting cancer cell proliferation [28]. Besides ER, other proteins involved in estrogen signals were assumed as PROTAC targets. Among them, the oncogene steroid receptor coactivator-3 (SRC-3) is essential to form an efficient ER transcription complex, by acting as a bridge between the ERα co-activator binding site and other co-activators. SRC-3 was shown to be overexpressed in breast tumor cells and to play a pivotal role in the occurrence and progression of endocrine resistance. Recently, a PROTAC (BY13, Figure 2) capable of efficiently degrading SRC-3 at submicromolar concentrations, was reported. Interestingly, it inhibited the proliferation of both wild-type and endocrine-resistant breast tumors, without appreciable toxicity in mice [29], thus revealing that different PROTAC approaches could be successfully exploited to overcome the serious problem of endocrine resistance in breast cancer.

It is worth noting that dual enzyme inhibitors could be selected as starting points to design new PROTACs. An interesting example of dual-targeting PROTAC was recently reported (compound 18c, Figure 2) [30]. The structure of the warhead was inspired by dual inhibitors in which both a phenol moiety and an imidazole ring were incorporated to target ERα and aromatase, respectively. This PROTAC succeeded in degrading both POIs and suppressing the proliferation of different breast tumor cells, including mutant strains, without cytotoxicity to normal cells [30]. Therefore, there is significant evidence that the PROTAC approach could be leveraged in the search for multitarget agents designed to treat different complex diseases, besides cancer, in which multiple pathogenic dysfunctions are implicated.

For many PROTACs reported so far, warheads were selected among small-molecule inhibitors or ligands which were already known as favorable starting points to ensure appreciable affinity of the final heterobifunctional ligand for a selected POI. Significantly, PROTAC-induced protein degradation often resulted in improved efficacy compared with parent inhibitors, especially in controlling tumor growth and, furthermore, in tackling drug resistance in both cancer and infectious diseases.

PROTACs could also degrade mutated proteins, regardless of site-specific mutations, if the warhead maintains its capacity to bind the selected POI. Mutations occurring in the binding sites of enzymes or other target proteins can reduce the affinity of effective drugs, leading to serious therapeutic complications for the treatment of several pathologies, especially cancer and infectious diseases. Protein degradation mediated by PROTACs could also overcome another type of chemoresistance, caused by the overexpression of drug target proteins which is often observed under long-term treatments with antiproliferative or antimicrobial drugs [4]. An interesting example is compound DGY-08-097 (Figure 2), which was synthesized as a degrader of the protease NS3/4A of hepatitis C virus (HCV), by incorporating the known drug telaprevir as warhead [31]. Telaprevir is a peptidomimetic compound endowed with an electrophilic α-ketoamide group capable of covalently binding a catalytic serine residue of the HCV protease. However, the therapeutic efficacy of this antiviral agent can be reduced by multiple resistance mechanisms that may be rapidly activated during the drug exposure or due to a preexisting NS3/4A polymorphism. Therefore, the incorporation of telaprevir in a PROTAC could overcome viral resistance to the drug, by specifically degrading the target enzyme, even in the presence of mutations. In the structure of DGY-08-097, the solvent-exposed pyrazine ring of telaprevir was conjugated with a novel tricyclic imide moiety endowed with high affinity for CRBN. The resulting PROTAC showed potent anti-HCV activity, by acting as an effective degrader of both wild-type NS3/4A protease and telaprevir-resistant NS3-V55A mutant [31].

On the other hand, although PROTACs show a significant potential as novel agents capable to tackle chemoresistance, it must be taken into consideration that further resistance mechanisms directed to PROTACs may also occur. In fact, it was demonstrated that prolonged exposure to these protein degraders could trigger resistance caused by mutations or deletion of UPS components [32]. Therefore, it is necessary to further investigate these critical aspects, which are fundamental to PROTAC functioning, to providing evidence for possible limitations, and to directing efforts to overcome them.

The conversion of a small-molecule enzyme inhibitor into a PROTAC can be exploited to modulate the selectivity towards different target proteins. For example, an interesting anticancer PROTAC, compound 1(*R*) (Figure 2), was obtained starting from foretinib, which is a promiscuous inhibitor capable of simultaneously targeting several receptor PTKs involved in tumor angiogenesis. Compound 1(*R*) was shown to be endowed with higher potency and selectivity than the parent compound, being able to degrade p38α kinase at nanomolar concentrations, without affecting other kinases which are inhibited by foretinib [33]. Interestingly, a cooperative binding of the warhead and E3 binder portions to form the ternary complex p38α/PROTAC/E3 ligase was suggested, to rationalize the markedly different capability of compound 1(*R*) to induce the degradation of p38α kinase as compared with other PTKs [33].

### 2.2. Cooperativity

Cooperativity can be often observed in the mechanism of action of PROTACs. In fact, the two functional moieties of a PROTAC can act synergistically to induce a close positioning of POI and E3 ligase, resulting in the formation of a highly productive ternary complex which can facilitate the transfer of ubiquitin to the target protein [4,34]. To this end, the linker can play a fundamental role, allowing the PROTAC molecule to assume a conformation favorable for the interaction POI/E3 ligase. Therefore, the affinity of both warhead and E3 binder toward their respective targets is necessary, but not always sufficient to achieve highly efficient protein degrading activity [4,5]. In fact, negative cooperativity could occur when the formation of the ternary complex gives rise to steric clashes, thus preventing the ubiquitination of the target, also in the presence of binding moieties endowed with high affinity for each protein.

The positive cooperativity can also be beneficial to counteract the so-called “hook effect”, which could occur at saturating doses of a PROTAC; under this condition, the formation of binary complexes of the heterobifunctional ligand with either the POI or E3 ligase could be favored, to the detriment of the formation of the ternary complex, consequently resulting in a lesser extent of protein degradation [4,6]. Therefore, during the development of a PROTAC as a potential drug, the stability and productivity of the POI/PROTAC/E3 ligase ternary complex must be carefully investigated, being a crucial determinant of the efficiency of PROTAC-induced target degradation. If the ternary complex is excessively stable, the PROTAC release could be prevented, and its catalytic feature could be compromised; in the extreme, a PROTAC that binds a POI covalently can be degraded along with its target by the proteasome. It was also reported that covalent binding of a heterobifunctional ligand to a protein could hinder substrate ubiquitination by E3 ligase or the recognition of the polyubiquitinated POI by the proteasome [4,35].

X-ray structural investigation of ternary complexes formed by PROTACs can provide a powerful tool to rationalize the impact of cooperativity on target protein degradation. An interesting example is given by GSK215 (Figure 2), a PROTAC directed to the focal adhesion kinase (FAK). FAK degradation induced by GSK215 resulted in a wide range of actions and greater effectiveness in blocking cell proliferation and tumor growth, compared with FAK inhibition [36]. Positive cooperativity in the ternary complex formation was elucidated by X-ray crystallography, showing that the heterobifunctional ligand promotes several productive interactions between the POI and VHL ligase, including effective ionic and hydrogen bonds [36].

### 2.3. Roles of Linker and E3 Binder Moieties in the Modulation of PROTAC Activity and Selectivity

It was demonstrated that both effectiveness and selectivity of a PROTAC can be tuned, not only on the basis of the features of the warhead, but also by appropriately selecting the other terminal moiety that is directed to recruit a specific E3 ligase as well as by varying the linker connecting these two portions [2,4,5,6,7,8,37]. E3 ligase binder incorporated in a PROTAC molecule can play a crucial role in the formation of a productive ternary complex and, consequently, in the early stage of PROTAC design, great attention is given to this moiety. In fact, appreciable differences in protein degrading efficacy were often observed between CRBN- and VHL-recruiting PROTACs bearing the same warhead directed to a given target [5]. However, the knowledge of human E3 ligases, which are a family of more than 600 enzymes, is still incomplete, especially about their tissue expression and, currently, the selection of a given E3 ligase for PROTAC design is generally empirical and still limited to a small number of these enzymes (e.g., CRBN, VHL, MDM2, IAP) [8].

The most widely exploited CRBN and VHL ligands are valuable tools for PROTAC design, due to the knowledge of their binding modes and high affinity to the target ligases [4]. A further benefit originates from the ubiquitous presence of these ligases and, consequently, from their capability to ubiquitinate a wide number of POIs in different tissues. However, the other side of the coin is that mutations and/or downregulation of UPS components could occur more frequently in ubiquitous enzymes, possibly leading to reduced effectiveness of PROTACs that recruit CRBN and VHL ligases [5,32].

The nature of the linker connecting the two binding moieties is another crucial determinant of PROTAC effectiveness [4,37,38]. In fact, modifications of the length, structure, flexibility, stereochemistry and linkage site of this portion can modulate both physico-chemical properties and biological activity of PROTACs, by playing a crucial role in determining ternary complex formation, target selectivity, membrane permeability and pharmacokinetics. Therefore, linker modifications are frequently exploited during PROTAC development to study their relationships with protein degradation and to optimize activity and selectivity profiles [37,38]. Flexible alkyl or polyethylene glycol (PEG) chains are very commonly used in the design of PROTACs, due to their adaptability and synthetic accessibility. Saturated or unsaturated alkyl chains are stable linkers that can be varied in length and flexibility; however, they are also highly hydrophobic and, therefore, they can be integrated or replaced by more hydrophilic chains, such as PEGs, to improve solubility in physiological environments [37]. The metabolic stability of a linker can be increased by replacing hydrolysable amide or ester groups with ether, alkyl or PEG-like chains or by introducing cyclic moieties, such as piperidine, piperazine or triazole rings, which can also modulate solubility and distribution of PROTACs [4,37].

Therefore, the optimization of the linker portion is a challenging and crucial step in PROTAC design and development. It is generally a computer-aided process, based on molecular docking simulations, molecular dynamics and machine learning models, but, especially in the early design phase, it requires intensive synthetic efforts to extensively study the relationships between different linkers and PROTAC degrading activity. In PROTAC design, early computational approaches are generally based on the knowledge of the structure of the selected POI as well as on the availability of small-molecule ligands, by generating potential PROTAC molecules which are then synthesized and experimentally evaluated. As in the case of conventional drug design, an iterative process of computational design, chemical synthesis and biological evaluation can result in PROTAC optimization in terms of potency, selectivity, and pharmacokinetics. Recently, it was also suggested that integrating artificial intelligence methods and conventional structure-based procedures might provide significant advances in the development of PROTACs [38]. On the other hand, it should be taken into consideration that, although numerous computational methods can effectively support the design and optimization of PROTACs, these processes are more complex than those of conventional small-molecule agents and successful results are less predictable.

## 3. Development of PROTACs Targeting PTPs

### 3.1. Potential of PTPs as Next-Generation Drug Targets

PTP superfamily comprises more than one hundred enzymes, which are divided into four main classes, based on their structural and functional features. Class I is the largest one and is characterized by a conserved Cys-based catalytic motif (H/V)C(X)5R(S/T), also named “PTP signature motif”, which is part of the catalytic phosphate-binding loop (P-loop). Class I PTPs are further divided into (a) classic tyrosine-specific PTPs, which are endowed with a catalytic pocket deep enough to specifically accommodate phosphotyrosine (pTyr) residues and catalyze only their dephosphorylation, and (b) dual-specific phosphatases (DUSP), which can also catalyze the hydrolysis of phosphoserine or phosphothreonine residues [13,14]. Classical tyrosine-specific PTPs include both cytosolic and transmembrane receptor-like proteins, among which several enzymes were identified and validated as drug targets, such as PTP1B, TCPTP, SHP2, CD45; alterations or deregulation of these PTPs were shown to play crucial roles in the pathogenesis of different human diseases, including diabetes, cancer, autoimmune and neurological disorders. Other noteworthy PTPs implicated in human pathologies, and considered potential targets for therapeutic interventions, belong to Classes II (low molecular weight PTP, LWMPTP) and III (CDC25) of the PTP superfamily [13,14].

However, the highly polar and conserved catalytic sites of these PTPs have posed challenges for drug design, because they require ligands bearing polar nonhydrolyzable pTyr-mimics, e.g., aryl phosphonates; these highly ionizable groups allow an effective binding to PTP catalytic sites, but, on the other hand, may be responsible for poor pharmacokinetics and scarce selectivity. In several cases, PTP inhibitors endowed with improved membrane permeability were obtained by replacing aryl phosphonate moieties with monoanionic carboxylic or heterocyclic pTyr-mimics as well as by introducing lipophilic substituents in their structures [15,39,40,41]. However, although numerous potent competitive inhibitors of several PTPs are known, their development as drug candidates was often interrupted. The design of inhibitors decorated with suitable substituents able to interact with subsites flanking the catalytic center, which are often lined with non-conserved residues, may lead to improved selectivity. A significant example is the non-catalytic secondary pTyr-binding site of PTP1B, which is the most extensively studied PTP so far; this site participates in substrate recognition and is lined with non-conserved amino acids, such as Tyr20, Arg24, Ala27, Phe52, Arg254, Met258, and Gly259 [42]. The presence of this non-catalytic pocket inspired the design and synthesis of a variety of bifunctional PTP1B inhibitors directed to both the catalytic site and the adjacent non-catalytic pocket, thus ensuring high affinity for the enzyme and improved selectivity over other PTPs [15,41,43].

Moreover, allosteric sites were individuated in certain PTPs, such as PTP1B and SHP2, thus prompting the design of non-competitive inhibitors endowed with better drug-like properties. In fact, the binding to these regions does not require polar pTyr-mimics and, in addition, significant divergences in the amino acid sequences of these noncatalytic sites were found. Therefore, allosteric PTP inhibitors potentially may display more favorable cell permeability and selectivity than catalytic site-directed inhibitors. Several non-competitive PTP1B inhibitors were reported in the last few years, thus renewing interest in the search for new agents targeted to this enzyme [21,44,45,46,47]. An allosteric site was individuated in a region of the enzyme located between helices α3 and α6: the binding of inhibitors to this hydrophobic site can prevent the closure of the catalytic WPD loop, which is necessary for the dephosphorylation mechanism, thus blocking PTP1B in its inactive conformation [44]. Another possible binding site for non-competitive inhibitors was found in a region of the protein surface between a β-sheet including Leu71 and Lys73, and a lipophilic pocket delimited by the loop Leu 204–Pro210; a β-strand consisting of only five amino acids connects this site to the PTP1B catalytic center and, consequently, the binding to the allosteric site could influence the conformation of the catalytic pocket, hampering the substrate recognition [45,46]. Significantly, among the few PTP1B inhibitors that entered clinical trials so far, two promising non-competitive inhibitors stood out, i.e., the natural aminosterol trodusquemine and its synthetic derivative DPM-1001 [21]. The possibility to design allosteric ligands also stimulated the search for inhibitors of other PTPs, among which SHP2 is a notable example [48,49].

Moreover, in the last few years, successful results achieved by means of the PROTAC approach suggested that this new mechanism of action could be leveraged for controlling PTPs implicated in human diseases. Compared with conventional inhibitors, the characteristics of PROTAC degraders potentially offer advantages to overcome some of the difficulties that have been encountered in the development of drugs directed at PTPs. In fact, the PROTAC approach gives opportunities to select a warhead in a more extended chemical space, since this moiety may be targeted to several regions of the protein surface, also distinct from the binding sites of competitive and non-competitive inhibitors. Moreover, selectivity could be more finely tuned by modifying both linker and E3 ligase-recruiting moieties.

### 3.2. Src Homology-2 (SH2) Domain-Containing Phosphatase 2 (SHP2)

Currently, heterobifunctional degraders directed to SHP2 are the most representative and advanced examples of PROTACs targeting PTPs. The availability of numerous inhibitors of the enzyme inspired the design of PROTACs which incorporated warheads derived from known SHP2 ligands.

SHP2 is a cytosolic Class I PTP and is encoded by the *PTPN11* gene. Besides the catalytic domain characterized by the PTP signature motif (H/V)C(X)5R(S/T), the structure of this enzyme includes two tandem terminal SH2 domains (N-SH2, C-SH2), which play a fundamental role in regulating the catalytic activity of the enzyme [50]. The crystal structure of SHP2 showed that, in the absence of a substrate, the N-terminal SH2 domain directly interacts with the catalytic pocket, preventing access to the active site and blocking the enzyme activity; this inactive closed conformation is defined as “autoinhibited structure”. The N-SH2 domain shows two separate surfaces, one for the binding to the PTP catalytic domain and one for the binding to phosphopeptides; when a tyrosine-phosphorylated protein binds to N-SH2, a conformational change in the region that interacts with the PTP domain is induced, consequently removing the block due to the autoinhibitory interaction and, thus, activating the catalytic function of the enzyme. Therefore, the N-SH2 domain functions as a molecular switch that is essential to regulate enzyme activity [50]. The C-terminal SH2 domain does not directly intervene in the regulation of enzyme activity but can recognize bisphosphorylated proteins in tandem with N-SH2 domain, thus increasing the dephosphorylating activity of SHP2 and contributing to the selectivity [50].

SHP2 acts as a negative regulator of insulin signaling and plays a critical role in promoting insulin receptor (IR) endocytosis [51]. Insulin receptor substrates 1 and 2 (IRS-1/2), which are phosphorylated by activated IR, undergo dephosphorylation by SHP2 and, consequently, the insulin signaling is disrupted. SHP2 deregulation was shown to be involved in insulin resistance in type 2 diabetes mellitus (T2DM); accordingly, SHP2 inhibition or liver-specific *PTPN11* deletion delayed IR endocytosis, enhanced insulin-activated AKT pathway and improved insulin sensitivity in mice [51]. Therefore, SHP2 inhibition or downregulation can be proposed as a useful strategy to treat diseases in which insulin resistance plays a crucial pathogenic role, such as T2DM, obesity, neurodegenerative disorders and cancer.

Moreover, SHP2 was recognized as an oncogenic PTP which is required for signal transduction in numerous kinase pathways implicated in tumor growth, survival and migration, such as RAS-MAPK-ERK, PI3K-AKT and JAK-STAT [52,53]. SHP2 dysregulations or mutations, especially in the N-SH2 and PTP domains, were observed in many human cancers, such as several types of leukemia and carcinomas, and were found to be positively correlated with tumor initiation and growth [54,55]. In addition, mutations of *PTPN11* are associated with Noonan syndrome, an autosomal dominant developmental disorder which is linked to high risk of developing malignancy [54]. Therefore, there is compelling evidence to support the identification of SHP2 as a bona fide oncoprotein; accordingly, SHP2 inhibition or *PTPN11* deletion were shown to prevent the progression of cancers driven by PTKs [56,57]. SHP2 is also involved in acquired resistance to anticancer drugs, and it was suggested that the activated gene *PTPN11* could be assumed as a biomarker for acquired chemoresistance in cancer [56]. Furthermore, SHP2 plays pivotal roles in T cell signaling, by participating to the programmed cell death 1 (PD-1) receptor signal transduction, which suppresses T cell activation and allows cancer cells to escape immune surveillance. Accordingly, SHP2 inhibition was shown to be a promising strategy to improve cancer immunotherapy by triggering antitumor immunity [53].

The capability of SHP2 to mediate signal transduction in several inflammatory pathways, often activated by cytokines and kinases, is also noteworthy, since it highlights the implication of this PTP in various inflammatory diseases, including cancer-related inflammation, neurodegenerative and metabolic disorders [58].

The oncogenic functions of SHP2 and its roles in mediating signals involved in tumor progression, immune response and inflammation make this PTP an attractive target for pharmacological intervention in several human diseases. A substantial number of compounds targeting SHP2 have been reported so far, which include both orthosteric and allosteric inhibitors [53,57,59]. In particular, the presence of several druggable pockets in the structure of SHP2, especially at the interface between PTP domain and N-SH2/C-SH2 domains, prompted the design of a variety of ligands, leading to the identification of potent drug-like allosteric inhibitors of the enzyme [57,59,60]. Several of them entered clinical trials as anticancer agents for the treatment of advanced solid tumors, as monotherapy or combined therapy with other drugs, especially PTK inhibitors [55,58,59].

**Figure 3:**
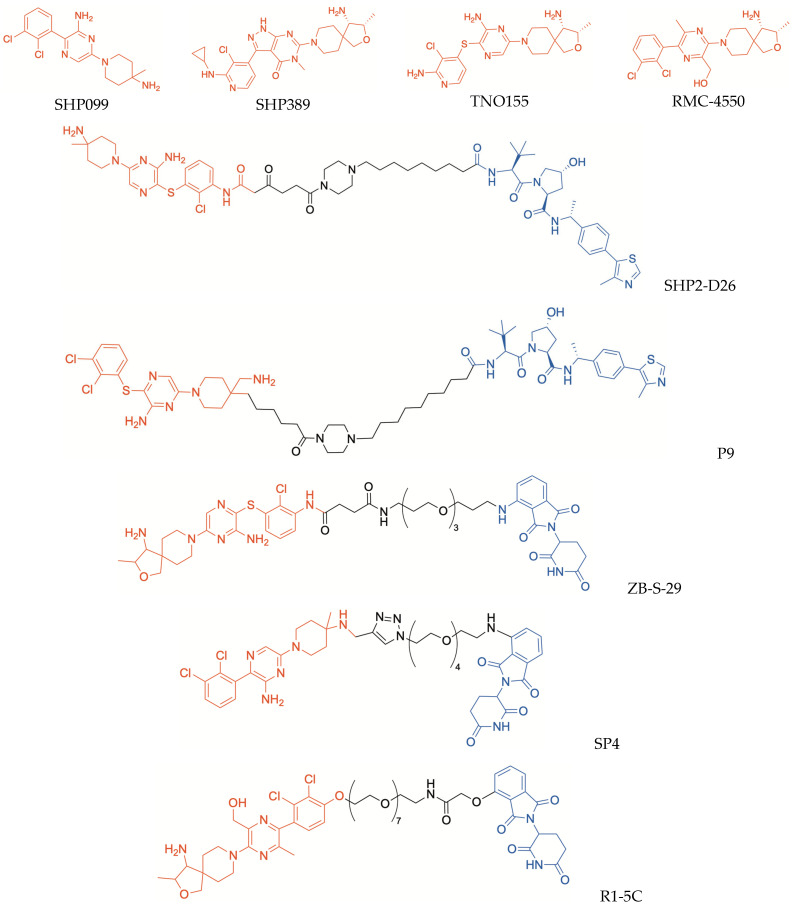
Structures of PROTACs targeting SHP2 (in red, POI-binding moiety; in blue, E3 ligase-binding moiety) and representative allosteric inhibitors which inspired their design.

Although allosteric inhibition of SHP2 proved to be a promising strategy to develop new anticancer candidates, it still requires a more complete understanding of the inhibition mechanisms triggered by occupation of different binding sites of the enzyme, as well as of the related SARs. In addition, mutated SHP2 forms, which are less sensitive to inhibitors, were found in several pathological conditions. Therefore, PROTAC-induced degradation of SHP2 was proposed as an alternative strategy to identify new agents capable of controlling this enzyme. Successfully, the first PROTAC directed to SHP2, SHP2-D26 (Figure 3), proved to be a potent degrader of the enzyme [61]. The design of this PROTAC was based on the X-ray structures of SHP2 co-crystallized with allosteric inhibitors SHP099 and SHP389 (PDB ID: 5EHR and PDB ID: 6MDC, respectively); these compounds can stabilize the “autoinhibited” conformation of the enzyme, by occupying a “tunnel-like” allosteric site located at the interface of the N-SH2, C-SH2, and PTP domains and establishing key interactions with all three domains [57]. Out of a series of potential PROTACs synthesized by modifying these inhibitors and conjugating them with a VHL-recruiting moiety via amide chains with different lengths, SHP2-D26 emerged as an excellent SHP2-degrading agent [61]. SHP2-D26 was shown to induce SHP2 degradation in both esophageal cancer KYSE520 and acute myeloid leukemia MV4;11 cell lines, in a rapid and dose-dependent manner, with DC_50_ values (concentration required to induce 50% degradation of the targeted protein) of 6.0 nM and 2.6 nM, respectively [61]. Moreover, in both cell lines, SHP2-D26 proved to be >30 times more potent than the parent inhibitor SHP099 in reducing the phosphorylation of ERK, thus impairing MAPK/ERK signaling pathway; it was also remarkably more potent than SHP099 in inhibiting tumor cell growth, with IC_50_ values ranging from 9.9 nM to 660 nM [61]. The potent antiproliferative activity of SHP2-D26, observed at concentrations significantly lower than IC_50_ values of the parent inhibitors, supported the initial hypothesis that SHP2 degradation could be an alternative strategy to achieve greater anticancer effectiveness compared with enzymatic inhibition. In addition, it is worth noting that the insertion of a piperazinyl ring in the linker moiety might be beneficial to achieve improved hydro-lipophilic balance, cell permeability and in vivo activity, like the above-reported ARV-110 and ARV-471.

Recently, another VHL-recruiting SHP2 PROTAC, also bearing a piperazinyl ring in the linker structure, was reported (compound P9, Figure 3) [62]. The warhead moiety of P9 is similar to that incorporated in SHP2-D26 but, in this case, the linker was inserted in the position 4 of the piperidinyl ring; thus, the warhead has a 180° flipped position compared with the previous PROTAC [62]. While alkyl and PEG-linkers generally produced unsatisfactory results, the insertion of an alkyl piperazinyl moiety was more beneficial. Compound P9 selectively degraded SHP2, without any significant effect toward other closely related PTP members, such as PTP1B, by inducing >95% SHP2 degradation at the concentration of 1 μM [62]. Moreover, it proved to inhibit the growth of tumor KYSE-520 cells, with an IC_50_ value of 0.64 µM; similar to SHP2-D26, this action was shown to be related to SHP2 degradation and to the consequent inhibition of the RAS/ERK1/2 signal transduction. Furthermore, in a murine xenograft model of KYSE-520 cells, the intraperitoneal administration of P9 provided a significant reduction in tumor growth and progression, without causing unwanted effects at the administered doses [62].

Other PROTACs targeting SHP2 were obtained by linking imide–CRBN binders to allosteric inhibitors of the enzyme. Thalidomide was conjugated with an analog of the allosteric inhibitor TNO155 (Figure 3), by means of succinamide–PEG chains of different lengths, leading to the identification of ZB-S-29 (Figure 3), which displayed highly efficient SHP2 degrading activity, with a DC_50_ value of 6.02 nM [63]. Modifications of the linker length resulted in decreased degrading action [63], once again highlighting the critical influence exerted by the tethering moiety on the activity profiles of PROTACs.

The allosteric inhibitor SHP099 was used as a starting point to synthesize another series of CRBN-recruiting PROTACs, characterized by a triazole ring, which was inserted in the linker through a classic “click chemistry” method [64]. Triazole-based linkers could be responsible for sustained action, being more stable in vivo than alkyl/PEG chains which, in contrast, are more prone to oxidative metabolization [37]. Among these triazole-containing PROTACs, compound SP4 (Figure 3) was identified as a potent SHP2 degrader. The treatment of HeLa cells with SP4 markedly inhibited cellular growth, at very low concentrations (IC_50_ = 4.3 nM), with 100-fold higher potency than the parent inhibitor; in these cells, SHP2 degradation resulted in impaired RES/MAPK signaling and increased apoptosis [64].

Analogously, another potent allosteric inhibitor of SHP2, RMC-4550 (Figure 3), was converted into a CRBN-recruiting PROTAC (R1-5C, Figure 3), by tethering its dichlorophenyl ring to talidomide through a PEG-linker; again, the length of this latter moiety proved to influence the protein degrading activity markedly, because shortening the linker resulted in reduction or complete loss of protein degrading capability [65]. Selective SHP2 depletion was caused in MV4;11 cells by treatment with R1-5C at 100 nM concentration; in addition, in KYSE-520 cells treated with this PROTAC, appreciable impairment of MAPK signaling was observed. Moreover, R1-5C noticeably inhibited both KYSE-520 and MV4;11 cell growth, with effectiveness comparable to that of the parent inhibitor RMC-4550. Considering its striking selectivity, it was proposed that R1-5C could also serve as a tool for investigating the oncogenic actions of SHP2 [65].

Interestingly, it was suggested that the co-existence of three allosteric sites adjacent to the SH2 and PTP domains (“tunnel”, “groove” and “latch” allosteric sites) could inspire the design of dual or trivalent PROTACs targeting SHP2 [59]; however, as noticed above, further elucidation of SHP2 functions is required to support the design of multitargeted agents.

In addition, it was proposed that dual PROTACs simultaneously directed to both SHP2 and a different target enzyme might achieve better efficacy, similar to combinations of SHP2 inhibitors with other drugs, especially PTK inhibitors [59]. A representative example is the combination of SHP2-D26 with the third generation EGFR inhibitor osimertinib, which elicited synergistic antiproliferative effects in animal models. When administered alone, SHP2-D26 moderately inhibited the growth of non-small cell lung cancer (NSCLC) mice xenografts, whereas its combination with osimertinib significantly reduced the growth of osimertinib-resistant xenografts [62]. These findings suggested that the simultaneous degradation of SHP2 and a PTK might provide an effective pharmacological tool to overcome acquired resistance, especially to PTK inhibitors, which is a serious concern in cancer treatment.

The promising outcomes obtained with PROTACs directed to SHP2 encouraged the search for new degrading agents to control other PTPs which are deregulated in different human diseases.

### 3.3. Protein Tyrosine Phosphatase 1B (PTP1B) and T-Cell Protein Tyrosine Phosphatase (TCPTP)

PTP1B is the prototypical member of the PTP superfamily, being the first PTP to be isolated and characterized. Established knowledge of the critical pathophysiological roles played by PTP1B in multiple cellular signals, especially involved in the development of insulin-resistance, leptin-resistance and carcinogenesis, supported the validation of this phosphatase as a molecular target for antidiabetic, antiobesity, and anticancer agents [15,21,39,66]. Protein tyrosine phosphorylation is fundamental for insulin signal transduction and regulation of cellular response to the hormone. The binding of insulin to its transmembrane receptor IR activates the intracellular β-subunit PTK, which is responsible for IR autophosphorylation and activation, with the subsequent recruitment and phosphorylation of IRS1/2 and downstream effectors (such as PI3K and AKT). PTP1B acts as a crucial negative regulator of insulin signaling, mainly in liver, skeletal muscle and brain, by dephosphorylating specific pTyr residues of IR and IRS, thus causing the attenuation of insulin cellular actions [14,67]. The dephosphorylating activity of this enzyme is essential also to regulate leptin signaling, through the dephosphorylation of the Janus 2 kinase (JAK2) associated with hypothalamic leptin receptors. The activation of leptin receptors in hypothalamus results in reduced appetite and increased energy expenditure and directly affects cellular insulin sensitivity [68,69]. The synergistic actions of insulin and leptin in the hypothalamus are fundamental for the energy homeostasis of the whole body [70,71]. Significantly, the control of both insulin and leptin signaling in the brain, by neuronal PTP1B, can elicit peripheral actions, in white and brown adipose tissues, pancreas, muscle, and liver, which are crucial to control body weight, glucose and energy homeostasis [70,71,72,73,74]. On the other hand, PTP1B expressed in muscle and liver mainly controls glucose homeostasis, without influencing weight gain induced by a high-fat diet [14,73,74,75,76]. Alterations in the expression or activity of PTP1B can result in a marked impairment of the signal transduction of both insulin and leptin, attenuating cellular response to these hormones. It was ascertained that in vivo PTP1B deficiency or silencing can result in enhanced signal transduction of both leptin and insulin, thus improving glucose homeostasis and providing resistance to diet-induced obesity, without causing abnormalities in growth or other vital functions [77,78]. In the liver, PTP1B deletion promotes insulin sensitivity mainly by restoring IRS-1-mediated PI3K/AKT/FoxO1 phosphorylation and inhibiting gluconeogenic enzymes [79,80]. Resistance to both insulin and leptin, resulting from PTP1B overexpression, is a characteristic condition found in complex human diseases, such as T2DM and obesity, and can also be an important risk factor for developing metabolic syndrome, cancer and neurodegenerative disorders. In fact, insulin plays pivotal roles in neuronal survival, synaptic plasticity, and memory/learning processes and it is well-established that insulin resistance has direct implications in the pathogenesis of Alzheimer’s disease (AD) [81]. Leptin receptors are also expressed in the hippocampus, and leptin signaling was shown to be involved in memory and cognitive processes. Consequently, leptin resistance has been recognized as another critical factor in the development of AD [82]. PTP1B deregulation can play a strategic role in linking resistance to insulin and leptin, neuronal stress, and inflammation; therefore, PTP1B inhibition could provide a novel disease-modifying strategy for the treatment of AD [83].

In addition, PTP1B was studied as a potential therapeutic target in cancer, since its implication in the development of specific tumor types, such as Erb2-induced breast cancer and its lung metastasis, was well documented. In mice with Erb2-induced breast cancer, PTP1B deficiency or inhibition resulted in reduced tumor progression and metastatization, along with increased apoptosis [84,85,86].

Therefore, PTP1B has been widely studied as a target for a variety of small-molecule inhibitors reported in the past three decades. However, similar to other PTPs, the search for PTP1B inhibitors as drug candidates proved to be challenging. The catalytic domain of PTP1B contains the above-mentioned “PTP signature motif”, including the conserved residues of Cys215 and Arg221, which are essential for the catalytic mechanism of dephosphorylation. At the edges of the PTP1B catalytic site, there are some critical loops involved in the substrate recognition and dephosphorylation: (i) the flexible WPD loop which, upon substrate binding, undergoes conformational changes to bring the catalytic acid Asp181 close to the hydrolysable phosphoester bond; (ii) the Q-loop, containing Gln262 which participates to the catalytic mechanism by correctly positioning a nucleophile water molecule; (iii) the YRD loop, containing Tyr46 which is critical for pTyr recognition [43,87,88]. Due to the highly conserved and polar nature of the PTP1B active site, effective small-molecule inhibitors targeting this region often exhibited scarce selectivity over other PTPs and/or inadequate bioavailability. The identification of the above-mentioned secondary non-catalytic aryl phosphate binding pocket, lined with less conserved residues and lacking in several other PTPs [42], prompted the rational design of potent selective bidentate inhibitors; however, these ligands were often highly polar compounds with poor cell permeability, which limited their further development. A more attractive alternative strategy is the design of inhibitors targeting allosteric sites that have been identified in the protein surface. As reported above, it was shown that structural features of these regions can provide opportunities for designing more selective ligands, endowed with more favorable drug-like properties, thus significantly increasing the promise of this PTP as drug target [19,21,89,90].

**Figure 4:**
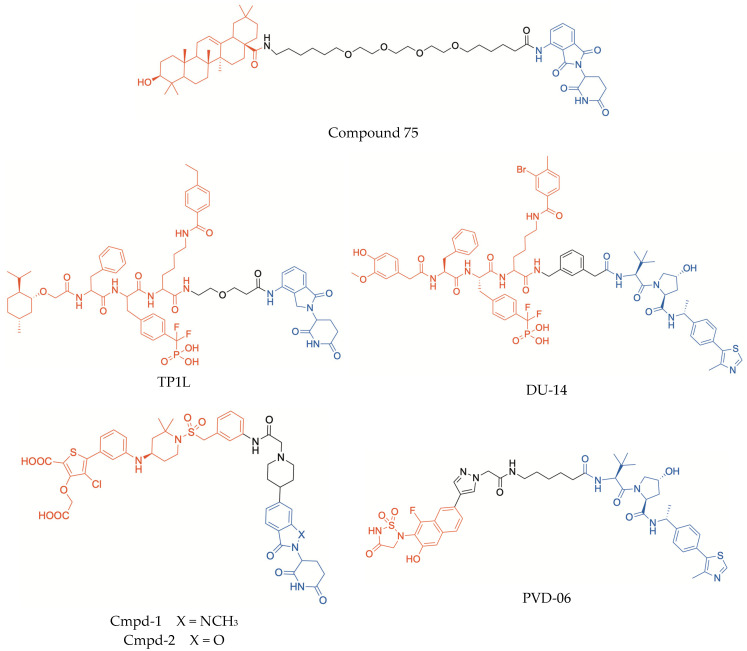
Structures of PROTACs targeting PTP1B and/or TCPTP (in red, POI-binding moiety; in blue, E3 ligase-binding moiety).

Furthermore, the PROTAC approach was recently exploited to design new antihyperglycaemic agents targeting PTP1B [91]. Three PTP1B inhibitors, i.e., the pentacyclic triterpenoids oleanolic acid and ursolic acid, and a synthetic benzoic acid derivative (ZINC02765569), were selected as warheads, according to their good PTP1B inhibitory properties (IC_50_ values ranging from 3 μM to 5 μM). On the basis of the X-ray structure of co-crystallized PTP1B/oleanolic acid (PDB: 1NL9), the solvent-exposed carboxylic group of each compound was conjugated with the CRBN binder pomalidomide by means of various alkyl/PEG chains, leading to the identification of an oleanolic acid derived-PROTAC (compound 75, Figure 4) which stood out as a long-term selective degrader of PTP1B [91]. In fact, compound 75 produced significant percentages of PTP1B degradation at submicromolar concentrations and, moreover, its degrading effect was shown to be more marked after 72 h than after 48 h (with DC_50_ values of 50 nM and 200 nM, respectively), thus proving to be a potential long acting antihyperglycaemic agent. Computational modeling suggested the formation of a stable ternary complex PTP1B/compound 75/CRBN, once again emphasizing the essential role of the linker length in determining favorable protein–protein interactions and consequent degrading effect [91]. Moreover, in HepG2 cells, compound 75 significantly improved the phosphorylation levels of downstream components of the insulin signal pathway, such as IRS-1, PI3K, and AKT, as a result of PTP1B degradation. It is plausible that this insulin mimetic effect may be responsible for the hypoglycaemic activity observed in the oral glucose tolerance test performed in KM mice [91].

Significantly, compound 75 is the first example of an antihyperglycaemic agent based on the PROTAC mechanism, which could pave the way to the design and optimization of other PTP1B degraders, by exploring more potent inhibitors as warheads, as well as diverse linkers and E3 ligase-recruiting moieties. Furthermore, considering that the pharmacological management of T2DM requires continuous lifetime therapies, the identification of new antidiabetic agents directed to this crucial enzyme and characterized by prolonged efficacy could be desirable. The pseudo-catalytic mechanism of action of PROTACs might be beneficial to achieve this goal, if degrading agents are not quickly metabolized and inactivated.

TCPTP is another PTP involved in the regulation of insulin signaling. It is a cytosolic Class I PTP, encoded by *PTPN2* gene and highly expressed in hematopoietic cells, which shares a high sequence similarity with PTP1B, especially in the catalytic domain (almost 80%) [92].

TCPTP functions as a negative regulator of insulin signal transduction, by dephosphorylating pTyr residues of IR β-subunit. TCPTP and PTP1B play cooperative nonredundant and temporally distinct roles in the regulation of IR activation and glucose homeostasis, in both peripheral tissues and central nervous system [14,93,94]. TCPTP overexpression can be induced by hyperleptinemia, which is typically observed in leptin resistant obese individuals; in turn, the enzyme attenuates leptin signaling by dephosphorylating the signal transducer and activator of transcription 3 (STAT3), a downstream effector of leptin signal which is phosphorylated and activated by JAK2 to mediate the transcription of genes involved in energy homeostasis [14]. In mice, deficiency of both PTP1B and TCPTP elicited additive effects by improving leptin sensitivity and glucose homeostasis [95]. It is worth noting that, in mice, homozygous TCPTP deletion was related to serious hematopoietic and immune disorders, whereas TCPTP^+/−^ heterozygous mice showed normal phenotype and life span. Therefore, the non-selective partial inhibition of both PTP1B and TCPTP was proposed as a possible strategy to improve the signal transduction of both insulin and leptin, without causing serious undesired effects [96,97,98].

Moreover, recently, the search for dual PTP1B/TCPTP inhibitors or degraders was stimulated by increased knowledge on the crucial roles of TCPTP in the regulation of immune system and tumor progression. TCPTP acts as a negative regulator of several PTK, such as JAK1 and JAK3, and transcription factors, such as STAT1 and STAT5, besides STAT3. An important consequence of controlling JAK/STAT pathways is the attenuation of interferon-γ (IFNγ) and interleukin-2 (IL-2) signaling, which can impair the recognition and elimination of cancer cells by T cells [98,99]. Therefore, TCPTP acts as a negative regulator of IFN signaling in immune and tumor cells, and, in fact, high levels of this enzyme were observed in human cancers that are resistant to current immunotherapy [100]. These findings supported the idea that TCPTP could be assumed as a pharmacological target in immuno-oncology. Increasing evidence indicated that TCPTP deletion or inhibition in tumor cells and/or immune cells can promote antitumor immunity and improve cancer responsiveness to immunotherapy. TCPTP deletion in T cells was shown to stimulate the activation, expansion, and survival of CD8^+^ T cells [101]. In a murine transplantable tumor model, the removal of TCPTP from tumor cells led to the amplification of IFNγ-mediated effects on antigen presentation and growth inhibition, thus improving the sensitivity of cancer cells to immunotherapy and blocking cancer progression [102]. In addition, in a murine model of triple-negative breast cancer, *PTPN2* deletion increased STAT1-dependent T cell recruitment and activation and, thus, promoted antitumor immunity [99].

PTP1B was also recognized as a negative regulator of several signaling pathways mediated by cytokines and T cell receptors and, in fact, its deletion can enhance expansion of T cells and cellular response to cytokines, improving the control of tumor growth [98,103]. Overall, since PTP1B and TCPTP can act coordinately to regulate diverse biological pathways, dual agents targeted to both these PTPs might be useful for therapeutic intervention in different diseases, particularly as novel agents capable of enhancing the anticancer actions of immune cells and inhibiting tumor progression.

TCPTP exists in two splice variants, TC45 (45 kDa) and TC48 (48 kDa); the former is the most abundant form (more than 95% of the total), on which the search for TCPTP inhibitors has been focused. The structure of TC45 displays high similarity degree compared with that of PTP1B, comprising the P-loop, the WPD loop and the E-loop in the catalytic region. In addition, helix α7, which was recognized as an allosteric switch in PTP1B, plays a similar role in TCPTP, proving to be fundamental for the efficient catalytic function of TCPTP [104]. However, the intrinsically disordered C-terminal regions of the two enzymes display some divergences, such as a RKRKR sequence in TCPTP, which functions as a localization signal necessary for the translocation of the enzyme in the nucleus [105].

In the last few years, several dual TCPTP/PTP1B inhibitors were reported [103,106,107]. They were generally designed as catalytic site-directed ligands, by including in their structures pTyr-mimics, such as difluoromethylphosphonate and thiadiazolidinone dioxide motifs, which can establish similar interactions with the active pockets of both enzymes. Recently, it was reported that a 1,2,5-thiadiazolidine derivative, ABBV-CLS-484 (AC484), able to potently inhibit both TCPTP and PTP1B at very low nanomolar concentrations, amplifies responsiveness of tumor cells to IFNγ, promotes T cell activation and impairs tumor growth in mice [103]. Interestingly, although the inhibitor was shown to act on both tumor cells and host immune system, it emerged that dual TCPTP/PTP1B inhibition in host immune cells is sufficient for eliciting antitumor effects [103]. It is significant that the dual inhibitor ABBV-CLS-484 entered a Phase I clinical trial (NCT04777994), in combination or monotherapy, in patients with locally advanced or metastatic tumors.

On the other hand, selective targeting TCPTP remains a very difficult task, due to the high structural and sequence identity with PTP1B [107]. However, these PTPs show differences in substrates and tissue expression. Considering that TCPTP levels are markedly higher in lymphoid and bone marrow tissues than in other sites, whereas PTP1B is more widely expressed and is present especially in tissues involved in glucose homeostasis, it was suggested that inhibitors more selectively directed to TCPTP might be potentially safer for cancer immunotherapy. At the same time, selective TCPTP inhibitors might serve as tools to further investigate the complex roles of this enzyme in tumorigenesis and immunity [108]. To increase the selectivity toward TCPTP, several PROTAC derivatives were designed starting from inhibitors of the enzyme.

A series of PROTACs were synthesized by exploiting a phosphonodifuoromethyl phenylalanine (F_2_PMP)-based PTP1B/TC-PTP dual inhibitor, endowed with high affinity for TCPTP and 7-fold selectivity over PTP1B, and conjugating this warhead with the CRBN-ligand lenalinomide through different linkers [108]. This SAR study led to the identification of compound TP1L (Figure 4) as a highly effective and selective degrader of TCPTP. In HEK293 cells treated with TP1L at 1 μM concentration, >95% TCPTP degradation was observed after 16 h of treatment, whereas PTP1B degradation was <5%. In these conditions, the value of DC_50_ of compound TP1L toward TCPTP was 35.8 nM, whereas the degrading effectiveness toward PTP1B was at least 110-fold lower. Interestingly, the capability of TP1L of inhibiting TCPTP is only 6-fold higher over PTP1B, suggesting that the marked selectivity in degrading TCPTP over the homologous enzyme could be due to the formation of a more productive ternary complex when the POI is TCPTP rather than PTP1B [108]. Therefore, the design of PROTACs could be a more promising strategy to obtain high selectivity toward TCPTP over PTP1B, compared with conventional inhibitors. In addition, no appreciable degrading effects towards other PTPs were observed at concentrations of TP1L up to 10 μM. In HEK293 cells, TP1L proved to amplify IFNγ signaling and to promote antigen presentation, by increasing the phosphorylation levels of both JAK1 and STAT1, in a dose-dependent manner. In Jurkat cells, TP1L was able to activate T cell receptor signaling and, moreover, in a carcinoma KB tumor/CAR-T cell co-culture system, it was shown to enhance CAR-T cell activation and to significantly promote their tumor killing efficiency [108].

Shortly before TP1L, a PROTAC acting as a dual TCPTP/PTP1B degrader, DU-14 (Figure 4), had been reported, which proved to degrade both these PTPs with similar potency [109]. Analogously to TP1L, compound DU-14 incorporated a (F_2_PMP)-based dual TCPTP/PTP1B inhibitor as warhead, which was conjugated with a VHL-recruiting moiety by means of a *m*-xylene ring. This unusual rigid and lipophilic linker proved to enhance the PROTAC degrading effect. However, differently from TP1L, in HEK293 cells, after 16 h treatment with DU-14, the degradation percentage of both target PTPs was similar (95% PTP1B, 92% TC-PTP, at 0.2 μM concentration), resulting in nearly identical DC_50_ values of 4.3 nM and 4.8 nM towards PTP1B and TC-PTP, respectively. In these cells, DU-14 proved to increase the phosphorylation levels of substrates of both PTPs, such as JAK1 and JAK2, and to amplify IFNγ signaling, thus promoting the recruitment and activation of T lymphocytes and the subsequent recognition and killing of tumor cells. Moreover, DU-14 inhibited the growth of colorectal tumors in mice [109].

It is worth noting that the (F_2_PMP)-based warheads of both TP1L and DU-14 (Figure 4) exhibit a high structural similarity which likely cannot justify the remarkably different capability of these PROTACs to induce proteasomal degradation of PTP1B. It is plausible that the different linkers and/or E3 ligase binders may play a predominant role in the formation of productive POI/PROTAC/E3 ligase ternary complexes, resulting in scarce PTP1B degradation in the case of TP1L, in contrast with the dual TCPTP/PTP1B degrading activity observed with DU-14. Moreover, it would be worth further evaluating the different impact of a genuine dual TCPTP/PTP1B degrader, as DU-14, and a more selective TCPTP degrader, such as TP1L, in animal models; this investigation might provide a valuable piece of knowledge about the therapeutic potential and possible undesired effects of degrading agents endowed with different potency toward these two PTPs.

A different dicarboxylic thiophene-based pTyr-mimic, which showed high affinity for the catalytic regions of both TCPTP and PTP1B, was incorporated into PROTAC molecules (Cmpd-1 and Cmpd-2, Figure 4), which displayed dual degrading activity toward both these PTPs [110]. The introduction of a *meta*-disubstituted phenyl ring in the position 5 of the thiophene scaffold allowed the insertion of the linker without compromising the binding to the PTPs. The linker was conjugated with an imide–CRBN binder, with the only difference in a benzoxazolone moiety in Cmpd-2, in place of the N-methylbenzimidazolone nucleus of Cmpd-1. This latter compound was shown to induce potent TCPTP/PTP1B degradation, both in cells and mice, with greater effectiveness than Cmpd-2 (DC_50_ values of 0.044 μM and 0.235 μM, respectively). In addition, Cmpd-1 exhibited high selectivity toward both TCPTP and PTP1B over other phosphatases [110]. Interestingly, the X-ray crystal structure of the complex TC-PTP/Cmpd-2 gave valuable insights into the key interactions between the warhead and amino acid residues of the catalytic region of the enzyme, also providing a rationalization of the capability of these PROTACs to function as degraders of both TC-PTP and PTP1B. Molecular modeling simulations indicated that each ligand could adopt the same binding pose in the catalytic sites of both PTPs, because the residues are highly conserved in these domains. Furthermore, a high-resolution structure of the complex DDB1-CRBN/Cmpd-1/TCPTP, obtained by means of cryo-electron microscopy and molecular dynamic simulations, revealed high plasticity at the POI-E3 ligase interface, indicating a dynamic nature of the PROTAC-induced cooperative ternary complex and suggesting that it might be useful investigating this aspect also in ternary complexes formed by other PROTACs [110].

A rationale approach to modulate degrading potency and selectivity can be based on the sequence differences in regions bordering the active sites of TCPTP and PTP1B, such as the E-loop and the C-terminal tail, also taking into account that these structural portions undergo conformational changes upon ligand binding and this might exert a remarkable effect on the formation of a productive ternary complex [106]. On this basis, the incorporation of a thiadiazolidinone dioxide−naphthalene warhead in a series of new PROTACs led to the identification of a selective TCPTP degrader, PVD-06 (Figure 4) [106]. The structure of the warhead was inspired by previously reported dual TCPTP/PTP1B inhibitors, such as ABBV-CLS-484, and was conjugated with the E3 binder moiety by means of different pyrazolyl acetamide linkers. The nature of both E3-recruiting and linker portions proved to exert a marked influence on the degrading effectiveness. In particular, the PROTACs of this series that incorporated a VHL binder were shown to be more potent degrading agents than analogs recruiting CRBN [106]. While some of the synthesized thiadiazolidinone dioxide-containing PROTACs caused also significant PTP1B degradation, PVD-06 stood out because of its remarkable selectivity (over 60-fold) toward TCPTP over PTP1B and other PTPs. PVD-06 was shown to induce significant TCPTP degradation in various cell lines, in a dose-dependent manner and, consequently, promoted T cell proliferation, amplified IFNγ signaling and inhibited melanoma B16F10 cell growth, thus exhibiting a potential for cancer immunotherapy [106]. Moreover, a computational model of the PTP/PVD-06/VHL ternary complex indicated that, while the thiadiazolidinone motif and the E3 binder of the PROTAC can interact with the P-loop and the VHL ligase, respectively, the E-loop of the enzyme is positioned at the interface between the two proteins, by assuming different conformations in TCPTP and PTP1B. When the target protein is PTP1B, the conformation of the E-loop was unfavorable, causing steric clashes that could prevent the formation of a productive ternary complex, thus rationalizing the observed selectivity of PVD-06 toward TCPTP [106].

## 4. Concluding Remarks and Prospects

In recent years, the potential of PTPs as novel drug targets has improved significantly, because it has become clear that the difficulties encountered in the development of therapeutic agents directed to these enzymes can be tackled by leveraging suitable medicinal chemistry approaches, which go beyond the design of competitive inhibitors. Two more relevant strategies have allowed PTPs to be definitely recognized as “druggable” targets: (a) allosteric inhibition, by means of small-molecule inhibitors designed as drug-like ligands targeting non-catalytic regions that have been individuated in the structures of certain PTPs, particularly SHP2 and PTP1B; (b) PROTAC-induced targeted degradation, which has already showed promise to identify new drug candidates directed to PTPs, although it has been exploited only in the last few years.

Currently, only few PTP-degrading PROTACs are known, which are directed to SHP2, PTP1B and TCPTP, and are still in the early steps of their development; however, activity data so far available showed that these agents can exert both in vitro and in vivo/ex vivo significative effects, in particular as potential anticancer and antidiabetic agents, thus demonstrating that PROTAC-mediated protein degradation could be a valuable strategy, alternative or complementary to conventional PTP inhibitors.

Remarkable results are related particularly to the opportunity to obtain agents endowed with improved effectiveness and selectivity, as compared with enzyme inhibitors. The reported PROTACs are generally potent agents capable of degrading target PTPs at very low concentrations, in nanomolar or submicromolar ranges. In several cases, the incorporation of known PTP inhibitors as warheads in PROTACs led to increased efficacy; significant examples are the SHP2 degraders SHP2-D26 and SP4, which exhibited more potent antiproliferative effects than the parent inhibitor SHP099. Moreover, PROTAC design was shown to be a promising strategy to overcome difficulties of obtaining selective ligands towards a given PTP, especially when the selected enzyme shares high similarity with other members of the PTP family, as in the case of TCPTP and PTP1B. In fact, the incorporation of warheads based on different dual TCPTP/PTP1B inhibitors in the PROTACs TP1L and PVD-06 led to selective degradation of TCPTP, with no appreciable effects toward PTP1B.

It is worth noting that the design of PROTACs targeting PTP1B and TCPTP started from active site-directed inhibitors; in the case of TCPTP, they were dual PTP1B/TCPTP inhibitors, based on the structural similarity of the catalytic regions of these enzymes. Whereas allosteric inhibitors of PTP1B are known and could be exploited as warheads for designing new PROTACs specifically directed to this PTP, the search for inhibitors of TCPTP was mainly focused on the active site of the enzyme, to the detriment of the identification of allosteric inhibitors. On the other hand, several allosteric binding sites were individuated in the structure of SHP2, promoting the identification of potent non-competitive inhibitors which were exploited as excellent starting points for the synthesis of PROTACs capable of specifically targeting this PTP.

Interestingly, comparing the selective TCPTP-degrading activity of TP1L with that of DU-14, a PROTAC which bears a similar warhead but acts as a dual TCPTP/PTP1B-degrading agent, highlighted the critical roles played by different E3 binders and/or linkers in determining the formation of productive ternary complexes that facilitate the selective degradation of a given target protein. The possibility to modulate PROTAC activity and selectivity via structural modifications of these moieties is very interesting from a medicinal chemistry point of view, because it could lead to the identification of novel ligands directed to PTPs in a chemical space wider than that explored so far, similar to other heterobifunctional degraders directed to different proteins. Moreover, a more extended knowledge of E3 ligases would be useful for the design of agents endowed with improved therapeutic potential, both in terms of efficacy and tissue selectivity. Specialized E3 ligases have been already identified in muscle and nervous tissues as well as in some tumor types [5]; further improvement of their knowledge would be desirable for designing new PROTACs aimed at controlling specific dysfunctions of PTPs in selected tissues.

Another interesting opportunity was suggested by the sustained insulin mimetic effects elicited by PTP1B degradation, provoked by the above-reported compound 75. It would be necessary to investigate other PROTACs specifically directed to PTP1B to verify whether the prolonged effect is related to the pseudo-catalytic protein degrading mechanism of these agents, as might be expected, and to evaluate both benefits and possible unwanted effects derived from this long-lasting activity. Based on these prospects, the way might be paved towards the development of a novel class of antidiabetic agents.

Notwithstanding these promising results and encouraging considerations, it must be taken into account that many aspects of the above-reported PTP-targeted PROTACs are still to be investigated, especially in vivo activity and toxicity. In vivo evaluation of few of them has been limited so far to intraperitoneal administration in mice, which is not suitable to predict bioavailability in humans. Challenging features of PROTACs, in general, can derive from their high molecular weights and large polar surfaces, which might result in limited membrane permeability and poor oral bioavailability [111,112]. PROTAC physico-chemical properties that are strictly related to oral bioavailability (e.g., lipophilicity) can be significantly affected by the nature of E3 binders; among them, CRBN-recruiting moieties were shown to offer better opportunities to achieve suitable drug-likeness, whereas MDM2, IAP and, in a lesser extent, VHL binders, appear to be less appropriate to design oral available PROTACs, due to their higher molecular weight and lipophilicity [112]. The structural features of linkers connecting the POI and E3 binder moieties were also shown to exert great influence, not only on the formation of productive POI/PROTAC/E3 ternary complexes, but also on diverse molecular parameters that are determinant for pharmacokinetics. Therefore, through medicinal chemistry efforts, the in vivo behavior of these heterobifunctional degraders could be improved to obtain more drug-like PROTACs, even endowed with oral bioavailability, as in some representative examples cited in this Perspective.

In addition, an important advance could be provided by the recent discovery of the pivotal role of the membrane cluster of differentiation 36 (CD36) in binding PROTACs and facilitating their cellular uptake [113]. It was shown that improved cell permeability and activity can be achieved by decorating the PROTAC linker, via a cleavable bond, with suitable moieties targeting CD36, thus promoting PROTAC uptake via CD36-mediated endocytosis [113]. Another innovative approach was proposed with in-cell click-formed proteolysis targeting chimeras (CLIPTACs); according to this strategy, CLIPTAC degraders are generated by the intracellular click reaction between two smaller cell permeable fragments, bearing a suitable warhead and an E3 ligase binder, respectively [114].

The development of PROTACs must also face other challenges related to tissue selectivity and off-target toxicity. Interestingly, considering that most PROTACs have been developed as potential anticancer agents, including degraders directed to PTPs mentioned above, different strategies were proposed to achieve selective release of active PROTACs into tumor cells, thus reducing toxic effects for normal tissues [115]. Among these, in analogy to prodrugs designed starting from small-molecule inhibitors, folate-caged PROTACs were synthesized by incorporating, via a hydrolysable linkage, a folate molecule into the prodrug; the rationale is based on the overexpression of folate receptor α in multiple types of tumors, which can promote the selective uptake of these prodrugs into cancer cells [115].

Other technologies, such as nanoparticles or other delivery systems, might be leveraged to improve water solubility, cellular permeability and selective tissue targeting of PROTACs, thus improving their pharmacokinetics and prompting clinical evaluation. Although all these strategies are promising and might significantly improve PROTAC therapeutic potential, the evaluation of both off-target effects and long-term complications remains a crucial aspect to be evaluated.

These prospectives could be applied to PROTACs directed to PTPs and prompt their further development. The promising results obtained so far encourage to leverage the PROTAC approach to obtain novel drug candidates for the treatment of human diseases characterized by overexpression or deregulation of specific PTPs, possibly extending the application of this strategy to other members of the PTP superfamily that have not been targeted by degrading agents yet. In addition, PROTACs could serve as chemical tools to further investigate the cellular roles of certain PTPs, as well as the effects derived from their degradation/downregulation. In fact, extending the knowledge of the functions of these enzymes could significantly improve our understanding of tyrosine phosphorylation-mediated cellular processes as well as of the consequences of the disruption of the phosphorylation balance in specific signaling pathways related to pathological conditions.
